# Quantum Zeno repeaters

**DOI:** 10.1038/s41598-022-19170-z

**Published:** 2022-09-12

**Authors:** Veysel Bayrakci, Fatih Ozaydin

**Affiliations:** 1 Faculty of Engineering and Natural Sciences, Isik University, 34980 Sile, Istanbul, Türkiye; 2 Institute for International Strategy, Tokyo International University, 1-13-1 Matoba-kita, Kawagoe, Saitama 350-1197 Japan; 3 Department of Information Technologies, Isik University, 34980 Sile, Istanbul, Turkey; 4 CERN, 1211 Geneva 23, Switzerland

**Keywords:** Quantum information, Quantum mechanics

## Abstract

Quantum repeaters pave the way for long-distance quantum communications and quantum Internet, and the idea of quantum repeaters is based on entanglement swapping which requires the implementation of controlled quantum gates. Frequently measuring a quantum system affects its dynamics which is known as the quantum Zeno effect (QZE). Beyond slowing down its evolution, QZE can be used to control the dynamics of a quantum system by introducing a carefully designed set of operations between measurements. Here, we propose an entanglement swapping protocol based on QZE, which achieves almost unit fidelity. Implementation of our protocol requires only simple frequent threshold measurements and single particle rotations. We extend the proposed entanglement swapping protocol to a series of repeater stations for constructing quantum Zeno repeaters which also achieve almost unit fidelity regardless of the number of repeaters. Requiring no controlled gates, our proposal reduces the quantum circuit complexity of quantum repeaters. Our work has potential to contribute to long distance quantum communications and quantum computing via quantum Zeno effect.

Long-distance communication is challenging in both classical and quantum domains. Because the effect of channel attenuation and various types of environmental noise on the transmitted information increases with distance. As a natural consequence, communication between two stations becomes impossible for great distances. In classical communications, this problem is solved by repeaters based on simple signal amplification. However, because measuring the state of a quantum system alters its quantum state and due to no-cloning theorem^1^, this idea is not applicable in quantum communications^2,3^. The solution in quantum domain, i.e., the idea of quantum repeaters is based on the so-called entanglement swapping (ES) process^4^, which require the implementation of multi-qubit controlled quantum logic gates. However, regardless of advances in quantum technologies, implementation of multi-qubit gates is more challenging than single-qubit gates. In this work, we ask whether ES can be realized without implementing multi-qubit gates. By designing a scheme based on quantum Zeno dynamics, we show that ES can be realized with almost unit fidelity only by implementing single-qubit gates and performing simple threshold measurements. Next, we show that ES can be extended to a series of stations toward building a quantum repeater system for enabling long-distance quantum communications.

## Entanglement swapping and quantum repeaters

**Figure 1:**
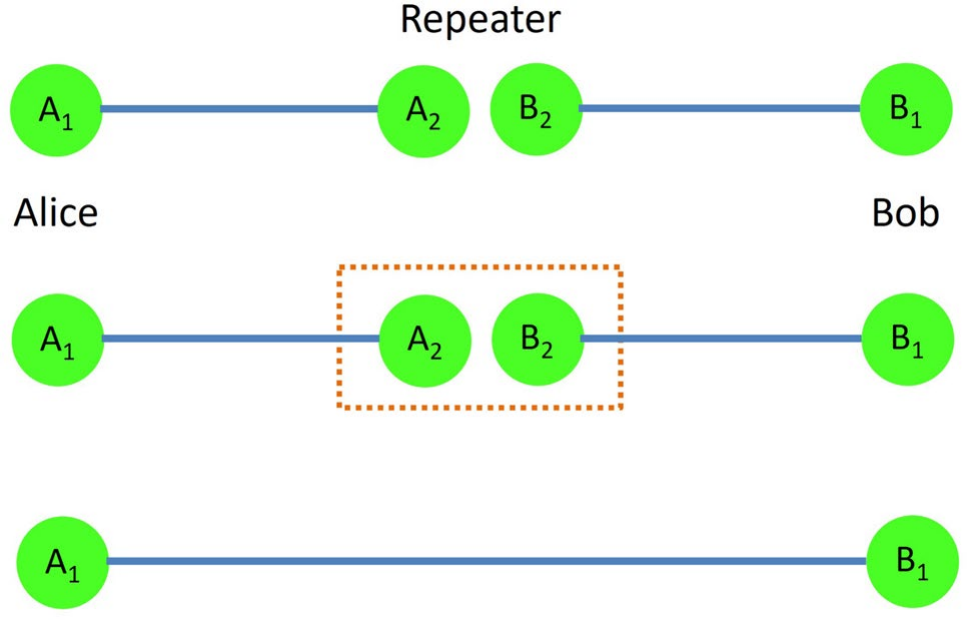
Illustrating the entanglement swapping procedure. Possessing two qubits, $A_2$ entangled with Alice’s qubit $A_1$, and $B_2$ entangled with Bob’s qubit $B_1$, Repeater performs operations and measurements on $A_2$ and $B_2$, leaving Alice’s and Bob’s qubits entangled.

As illustrated in Fig. 1, the idea of entanglement swapping^4^ can be summarized as follows. Consider that the distance between two parties, Alice and Bob is beyond the limits of sharing entanglement reliably, and that the half of the distance is within the limits. Placing a repeater station in the middle, Alice prepares a pair of entangled particles and sends one particle to the station. Bob repeats the same procedure. Then the repeater station applies local controlled-operations on the two particles it possesses, and the other two particles possessed by Alice and Bob become entangled.

In details, let a system of four qubits in the state $|\Psi _{A_1 A_2 B_2 B_1 }\rangle$ be initially shared among Alice, Repeater, and Bob; each qubit denoted as $A_1$, $A_2$ and $B_2$, and $B_1$, respectively, in the computational basis as

1 $$\begin{aligned} |\Psi _{A_1 A_2 B_2 B_1 }\rangle = \frac{|0_{A_1} 0_{A_2} \rangle + |1_{A_1} 1_{A_2} \rangle }{\sqrt{2}} \otimes \frac{|0_{B_2} 0_{B_1} \rangle + |1_{B_2} 1_{B_1} \rangle }{\sqrt{2}}. \end{aligned}$$

**Figure 2:**
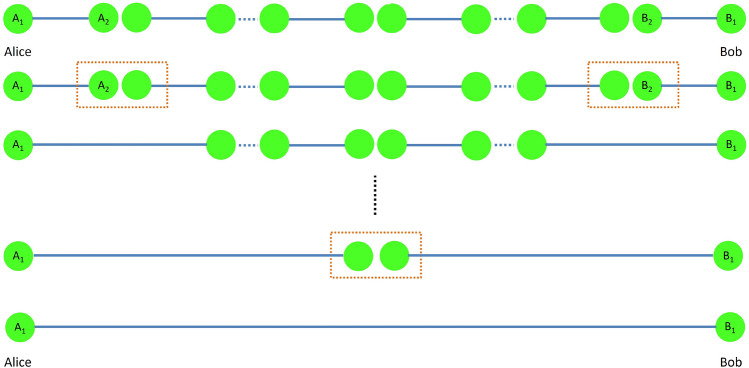
Extending the entanglement swapping procedure in Fig. 1 to long distances with many repeater stations in between.

**Figure 3:**
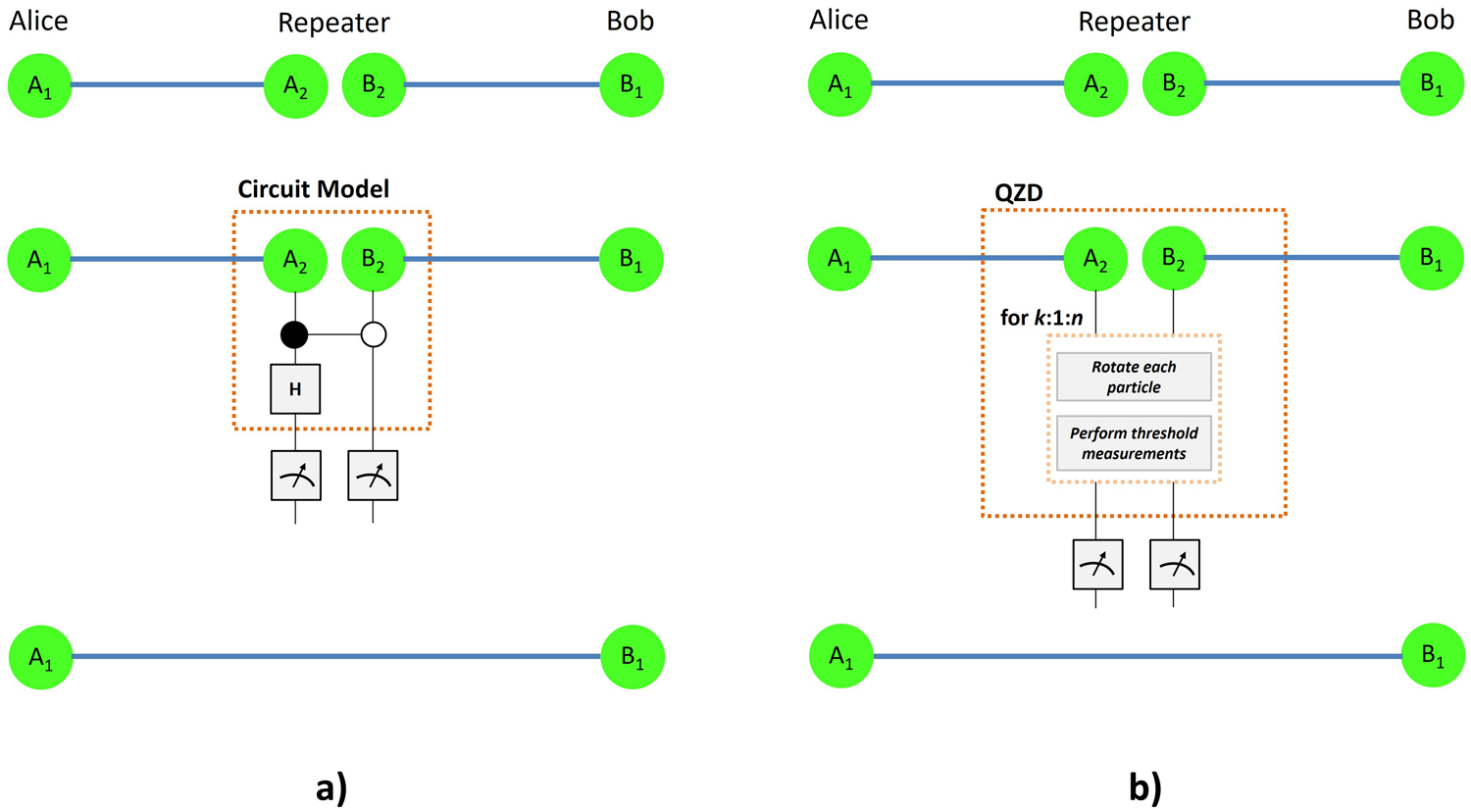
Entanglement swapping procedure via (**a**) the usual circuit model consisting of a CNOT and a Hadamard gate, (**b**) the proposed QZD strategy consisting of only single qubit rotations and simple threshold measurements, requiring no controlled two-qubit gates.

**Figure 4:**
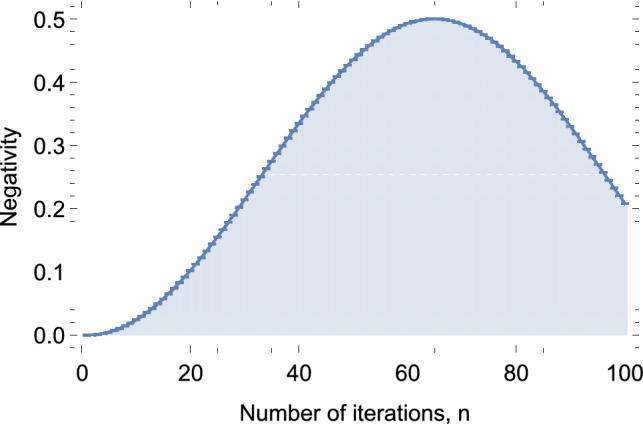
Negativity of the state obtained after *n* rotate-measure iterations of QZD for realizing a single entanglement swapping as in Fig. 3b.

**Figure 5:**
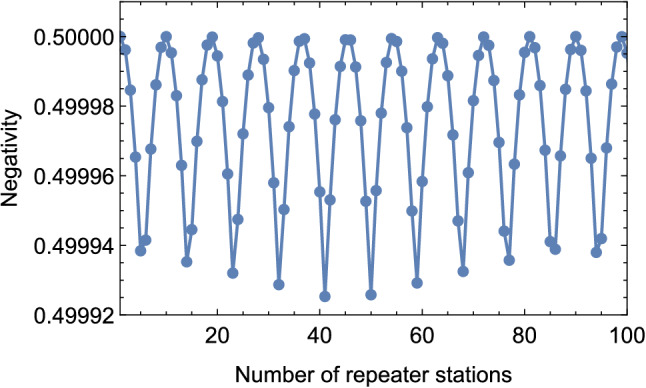
Negativity of the obtained state after ES over a hundred repeater stations.

**Figure 6:**
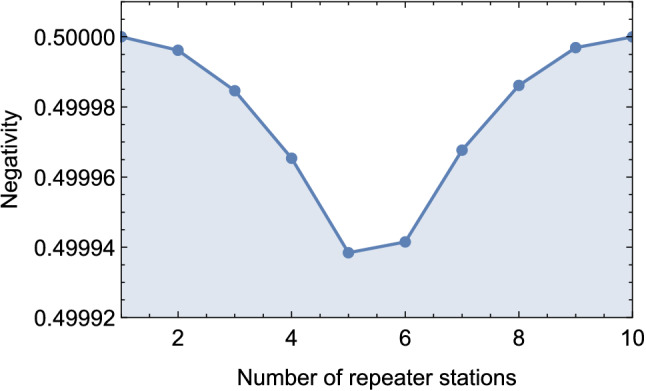
Negativity of the obtained state after ES over a few repeater stations for demonstrating one of the periodic turning points.

**Figure 7:**
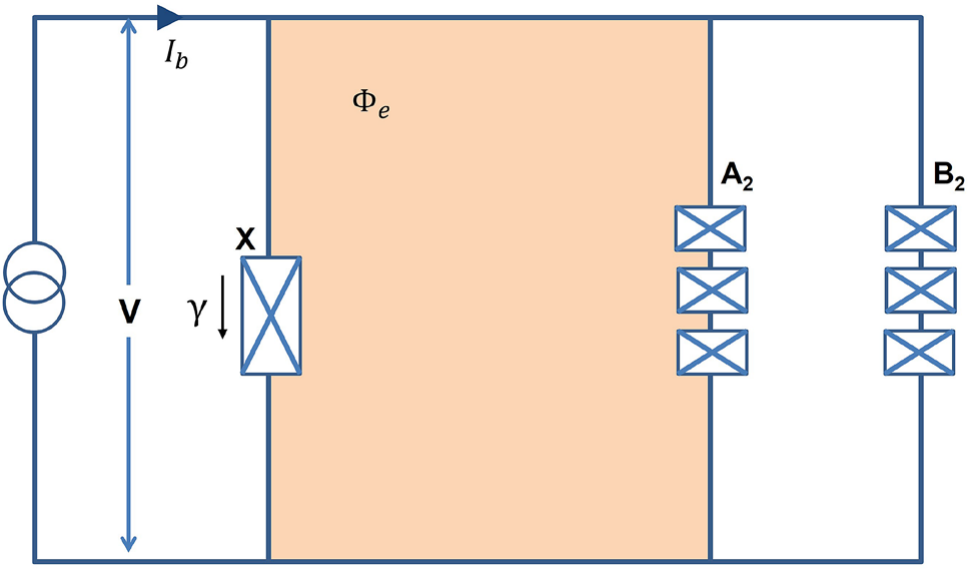
The Josephson-junction circuit with flux qubits designed by Wang et al.^47^ for implementing the J-measurement. Each flux qubit labeled as $A_2$ and $B_2$ consists of three small junctions. $\Phi _e$ is the flux of the external magnetic field threading the loop connecting the larger junction “0” and $A_2$ qubit. Following the implementation of QZD, qubits $A_2$ and $B_2$ will be measured in *z*-basis, leaving qubits $A_1$ and $B_1$ entangled, i.e., the entanglement swapping procedure is completed.

A two-qubit controlled-NOT (CNOT) gate is applied to qubits $A_2$ and $B_2$ as the control and target qubits, respectively, followed by a Hadamard gate on $A_2$. Then qubits $A_2$ and $B_2$ are measured in *z*-basis, yielding results $\{|i\rangle \}_{i=0,1}$. Measurement results are communicated through classical channels with Alice and Bob. Applying one of the single qubit operations $\{ I,\sigma _x,\sigma _z\}$ accordingly, Alice and Bob are left with and entangled pair of qubits,

2 $$\begin{aligned} |\Psi _{A_1 B_1 }\rangle = \frac{|0_{A_1} 0_{B_1} \rangle + |1_{A_1} 1_{B_1} \rangle }{\sqrt{2}}, \end{aligned}$$

where *I* is the two-dimensional identity operator, and $\sigma _x$ and $\sigma _z$ are the Pauli operators.

Extending the entanglement swapping process over a commensurate number of repeaters, Alice and Bob can share an entangled state, as shown in Fig. 2, regardless of how long the distance is between them^5^. This makes the quantum repeaters essential for long distance quantum communication and quantum Internet, attracting an intense attention from both theoretical and experimental points of view.

In addition to the photon loss, various types of noise pose a challenge. Through a nested purification protocol Briegel et al. designed a quantum repeater mechanism to overcome the exponentially scaling of error probability with respect to the distance^6^, and enabling reliable communication despite the noise in the channel allows quantum key distribution (QKD) over long distances with unconditional security^7^. Childress et al. considered active purification protocol at each repeater station for fault tolerant long distance quantum communication and proposed a physical realization of the protocol based on nitrogen-vacancy (NV) centers^8^. It was predicted that the hybrid design of van Loock et al. based on light-spin interaction can achieve 100 Hz over 1280 km with almost unit fidelity^9^. Generating encoded Bell pairs throughout the communication backbone, the protocol of Jiang et al. applies classical error correcting during simultaneous entanglement swapping and can extend the distance significantly^10^. Yang et al. have proposed a cavity-QED system which does not require the joint-measurement^11^, and showed that entanglement swapping can enable entanglement concentration for unknown entangled states^12^.

The light-matter interaction at repeater stations mainly for storing the quantum information in matter quantum memories was believed to be necessary, which makes the physical realization challenging. However, designing an all-photonic quantum repeaters based on all flying qubits, Azuma et al. have showed that it is not the case, making a breakthrough in bringing the concept of quantum repeaters to reality^13^.

Though requiring quantum memories at repeater stations, using spontaneous parametric downconversion sources, the nested purification^14^ and fault-tolerant two-hierarchy entanglement swapping^15^ have been experimentally demonstrated. Entangling electrons and nuclear spins through interactions with single photons, Kalb et al. have generated copies of remote entangled states towards quantum repeaters^16^. Recently, the idea of building quantum repeaters without quantum memory was experimentally demonstrated recently by Li et al. using linear optics^17^. For a thorough review of recent advances in quantum repeaters, please refer to ref.^18^.

Implementing the entanglement swapping procedure at each repeater station requires the realization of controlled two-qubit operations in the usual circuit model. Regardless of the technology and type of physical particles used as qubits, realizing two-qubit logic operations is a bigger challenge than single-qubit operations. Hence, in this work, we ask whether entanglement swapping can be implemented without controlled two-qubit operations, which could bring the quantum repeaters closer to reality. We consider quantum Zeno dynamics for serving this purpose. Beyond practical concerns towards long distance quantum communication and quantum Internet, building quantum repeaters based on quantum Zeno dynamics have potential to contribute to fundamentals of quantum entanglement.

### Quantum zeno dynamics

The quantum Zeno effect (QZE) can be described as follows^19,20^. If a quantum system in state $|e\rangle$ initially (at $t=0$) evolves under Hamiltonian ${\hat{H}}$, the probability of finding it in the same state, i.e. the *survival probability* at a later time (at $t>0$) is given as

3 $$\begin{aligned} p(t) = \left| \left\langle e \left| \exp \left( -{i\over \hslash } {\hat{H}} t\right) \right| e\right\rangle \right| ^2. \end{aligned}$$

Assuming the Hamiltonian ${\hat{H}}$ with a finite variance $\langle V^2 \rangle$ and considering short times, the survival probability is found to be

4 $$\begin{aligned} p(t) \approx 1- \frac{1}{\hslash ^2} \langle V^2 \rangle t^2. \end{aligned}$$

Now, let us assume ideal projective measurements on the system at intervals $\tau$. For $1/\tau \gg \langle V^2 \rangle ^{1 \over 2} / \hslash$, the survival probability is

5 $$\begin{aligned} p^n(\tau ) = p(t = n \tau ) \approx \exp \left[ - \frac{1}{\hslash ^2}( \langle V^2 \rangle \tau ) t \right] . \end{aligned}$$

In other words, the evolution of the system from the initial state slows down with $\tau$. What is more, for $\tau \rightarrow 0$, the survival probability *p*(*t*) approaches 1, which is widely considered as freezing the evolution of the system, such as in freezing the optical rogue waves^21^ and quantum chirps^22^. It was also shown that the frequent measurements can be designed for accelerating the decay of the system, which is also known as the quantum anti-Zeno effect (QAZE)^20^. Introducing a carefully designed set of quantum operations between measurements, QZE can be used to drive the a quantum system towards a target state, which is also known as the quantum Zeno dynamics (QZD).

One of the early experimental evidences of QZE was that in the the RF transition between two $^9\text {Be}^+$ ground state hyperfine levels, collapse to the initial state was observed if frequent measurements are performed^23^. QZE has been studied for slowing down the system’s evolution in Bose-Einstein condensates^24^, ion traps^25^, nuclear magnetic resonance^26^, cold atoms^27^, cavity-QED^28–30^ and large atomic systems^31^. QZE is being considered in various fundamental concepts. For example, it has been demonstrated in *PT*-symmetric systems in symmetric and broken-symmetric regions^32^. Quantum heat engines have been attracting an increasing attention in quantum thermodynamics^33,34^, and Mukherjee et al. has recently discovered the advantages of anti-Zeno effect in fast-driven heat engines^35^. Qiu et al. have showed that by controlling a proximal electron spin of a NV center, it is possible to realize QZE in the $^{13}\text {C}$ nuclear spin^36^. For studying QZE and QAZE, Ai et al. have obtained the effective Hamiltonian without rotating wave approximation and identified cases where QAZE disappears and only QZE remains^37^. Ai et al. have also studied QAZE for measurement-induced enhancement of the spontaneous decay for a two-level subsystem embedded in a three-level atom without wave function reduction^38^. Developing a framework for QZE of any system-environment model in the weak coupling regime, Chaudhry showed that the effective lifetime of a quantum state depends on the overlap of the spectral density of the environment and a generalized filter function which depends on the system-environment Hamiltonian^39^. QZE and QAZE in weak and strong coupling regimes^40–42^ and nonuniform couplings in a spin model^43^, as well as optimal^44^ and non-selective^45^ projective measurements have been studied in detail. Very recently, Majeed and Chaudhry studied two-level systems in both weak and strong coupling regimes to illustrate non-trivial effects of QZE and QAZE^46^.

An interesting application of QZD in quantum information and computation is creating entanglement between two initially separated qubits by applying single-qubit operations and performing simple threshold measurements in an iterative way, without requiring a CNOT gate^47^. Reducing the quantum circuit complexity by removing the controlled operations is promising for physical realizations. In a similar vein, recently, the activation of bound entanglement was shown to be enabled via QZD based on single particle rotations and threshold measurements^48^, which requires several three-level controlled operations, bound entangled states and classical communications otherwise in the original activation proposal by Horodecki et al.^49^. Quantum Zeno effect has been studied for generating multi-partite entanglement as well^50,51^, which is one of the most important problems attracting serious efforts in quantum science and technologies^52–56^.

## Results

Our QZD proposal for entanglement swapping starts with the joint system of two Bell states as in Eq. (1), described by the density matrix $\rho _{A_1 A_2 B_2 B_1}$. For simplicity, we set $\theta =\pi /180$ for the single qubit rotation operator presented in Eq. (8), and through numerical studies we find the threshold measurement operators to be $J_1=|1\rangle \langle 1|\otimes |1\rangle \langle 1|$ with $J_0 = I-J_1$ in accordance with Ref.^47^, as defined in Eq. (9). First, let us examine the case of a single iteration, i.e., $n=1$ in the procedure illustrated in Fig. 3b. After a single rotate-measure iteration on qubits $A_2$ and $B_2$ as described by Eqs. (10) and (11), respectively, we proceed with the final measurement in $z-$basis. The evolution of the system depends on the results of *z*-basis measurement in each iteration. Through numerical simulations, we find that proceeding with the case finding $|0\rangle \otimes |0\rangle$ state for simplicity, ES is realized after a sufficient number of iterations. However, more complex simulations might reveal cases where different *z*-basis measurement results can achieve the protocol with higher performance, such as in terms of a smaller number of iterations. Finding $|0\rangle \otimes |0\rangle$, the system of two qubits $A_1$ and $B_1$ is found approximately in the state

6 $$\begin{aligned} \displaystyle \rho ^1_{A_1 B_1} \!=\!\! \left( \begin{array}{cccc} \ \ \ 0.9993 &{} -0.0174 &{} -0.0174 &{} 0.0003 \\ -0.0174 &{} \ \ \ 0.0003 &{} \ \ \ 0.0003 &{} 0 \\ -0.0174 &{} \ \ \ 0.0003 &{} \ \ \ 0.0003 &{} 0 \\ \ \ \ 0.0003 &{} 0 &{} 0 &{} 0 \\ \end{array} \right) , \end{aligned}$$

where the superscript denotes the number of iterations performed.

To find after how many iterations we should end the QZD procedure, we run the simulation one hundred times and end the procedure at *n*th run (consisting of *n* iterations) with $n=1,2,...,100$. After each, we calculate the negativity of the resulting state $\rho _{A_1 B_1}$, which we plot in Fig. 4. Our simulation shows that within this setting, the resulting state after $n=65$ iterations (and after a $\sigma _x$ by Alice following the *z*-basis measurement result) is approximately

7 $$\begin{aligned} \displaystyle \rho ^{65}_{A_1 B_1} \!=\!\! \left( \begin{array}{cccc} \ \ \ 0.4993 &{} \ \ \ -0.0174 &{} \ \ \ 0.0193 &{} \ \ \ 0.4993 \\ -0.0174 &{} \ \ \ 0.0006 &{} -0.0006 &{} -0.0174 \\ \ \ \ 0.0193 &{} -0.0006 &{} \ \ \ 0.0007 &{} \ \ \ 0.0193 \\ \ \ \ 0.4993 &{} -0.0174 &{} \ \ \ 0.0193 &{} \ \ \ 0.4993 \\ \end{array} \right) , \end{aligned}$$

with negativity $N(\rho ^{65}_{A_1 B_1})=0.4999$ , calculated via Eq. (12). The fidelity of this state to the maximally entangled Bell state in Eq. (2) is found to be $F(\rho ^{65}_{A_1 B_1}, |\Psi _{A_1 B_1 }\rangle ) := \langle \Psi _{A_1 B_1}| \ \rho ^{65}_{A_1 B_1} \ |\Psi _{A_1 B_1} \rangle =0.9986$. This result shows that the entanglement swapping can be implemented with almost a unit fidelity by QZD, i.e., only through single qubit rotations and simple threshold measurements, without requiring any controlled operations, reducing the complexity of quantum repeaters significantly in terms of controlled two-qubit operations.

Next, we extend the QZD-based ES to a series of repeater stations. We consider the state $\rho ^{65}_{A_1 B_1}$ obtained from the first ES to be one of the two states of the second ES and the other being a maximally entangled state equivalent to $|\Psi _{A_1 B_1}\rangle$. The obtained non-maximally entangled two-qubit state in the second ES will then be considered for the third ES with a maximally entangled state, and so on for enabling long-distance quantum communication via quantum Zeno repeaters (QZR). At the first glance, it might be expected to obtain the new state with decaying negativity at each ES, vanishing with increasing distance, i.e., the number of repeater stations. However, this is not the case, demonstrating the strength of our proposed QZD. The negativity of the state obtained from each ES exhibits an oscillation. For example, after it decreases to 0.499938 in the fifth ES, it increases to 0.499942 in the sixth. We plot the negativity values of the states obtained over 100 repeater stations in Fig. 5. To provide a clearer presentation of the turning point of the negativity, we also plot the results for the first 9 states in Fig. 6, and provide the corresponding density matrices in the Supplementary Material.

## Discussion

### Contributions

A major contribution of the proposed quantum Zeno repeaters (QZR) is to reduce the quantum circuit complexity of repeaters in terms of controlled multi-particle operations as illustrated in Fig. 3a, which is more challenging than single particle operations in any technology in principle. Because our QZR protocol can be extended to multi-level particles, this reduction would be even more significant than the case of qubits. However, beyond practical concerns for reducing the quantum circuit complexity, we believe showing that quantum repeaters can be realized via quantum Zeno dynamics contributes to our understanding of quantum entanglement and measurements.

### Drawbacks

One of the drawbacks of our protocol is that under ideal conditions except for the attenuation in the channel which requires the repeaters in the first place, not exactly but almost unit fidelity can be achieved. However, over 0.998 fidelity can be tolerated in physical realizations especially given that the fidelities will decrease in both approaches. A more serious drawback could be the increased latency. Repeaters based on the standard circuit model requires the implementation of only two logic operations -though one being the controlled multi-particle operation. Our protocol requires the implementation of several single-particle operations and simple threshold measurements, instead. This would take a longer time depending on the physical realization, introducing a higher latency, which might not be desired especially considering on-demand systems and designs without quantum memory.

### KPI and related issues

The slight increase in the negativity does not violate the monotonicity of entanglement measure since a single entangled state with negativity $\approx 0.5$ is obtained out of two entangled states with total negativity $\approx 0.5$. The reason we prefer the negativity entanglement measure as the key performance indicator over the fidelity is as follows. In each ES, the resulting state is close to one of the four Bell states, which are equivalent under local operations and classical communications^1^. Hence, rather than finding which Bell state it is the closest to and then calculating the fidelity each time, for simplicity, we chose to calculate the negativity which is invariant under Pauli operators that the parties can apply to convert one Bell state to another. Note that while our QZD-based ES protocol requires 65 iterations in the first repeater, next repeaters might require a different number of iterations. Our simulation picks the best number for each repeater station and the presented results are based on the the best outcomes.

### Physical realization and robustness

For the physical realization of our QZD protocol, we consider the superconducting circuit proposed by Wang et al.^47^ where the threshold measurements can be implemented by Josephson-junction circuit with flux qubits, which is also summarized in the “Methods” section. In the same work, physical imperfections were also analyzed by considering a possible deviation from the rotation angle $\theta$ in each iteration. It was found that in the case of several iterations, the impact of the deviations is minimized, implying the robustness of the protocol. Because our protocol follows a similar rotate-measure procedure with many iterations, we consider a similar inherent robustness, too. Apart from the attenuation in the channel, we have studied our protocol under ideal conditions. However, because QZE has been mostly considered for protecting the system from noise induced by interactions with the environment, it would be interesting as a future research to design a QZD protocol with an inherent error-correction mechanism.

### Probability of success and future work

The probability of a *successful* *J*-measurement, $p_s$, i.e., the system is projected to the desired subspace slightly depends on the rotation angle $\theta$. However, because the state of qubits $A_2$ and $B_2$ keep evolving in each iteration, the $p_s$ value keeps changing, though within a very small range. Therefore, numerical results obtained through simulation might be more applicable than analytical expressions for the $\theta$ dependency of successful QZD iterations. For $\theta \approx \pi / 180$ or smaller, $p_s$ starts at around 3/4 and then achieves almost unity in next iterations. It is straightforward to find that the probability of success slightly decreases for larger $\theta$, for example, $p_s \approx 0.98$ for $\theta =10 \pi / 180$. However, this is not the case for the final *z*-basis measurement after the last iteration. Within the current set of parameters including very small $\theta$, following 65 rotations, the probability of finding $A_2$ and $B_2$ qubits in the $|0\rangle |0\rangle$ leaving $A_1$ and $B_1$ qubits in almost a maximum entangled state turns out to be approximately 1/3, while other possible outputs leave them in a non-maximally entangled state with negativity around 0.25. At this point, choosing a greater $\theta$ gives rise to the following trade off. A decent result can be achieved in less number of iterations, with the drawback of a smaller success probability of the final *z*-basis measurement. For example, 33 iterations are sufficient for the best result with $\theta =10 \pi / 180$, though the final *z*-basis measurement’s success probability turns out to be $\approx 0.21$. Note that throughout numerical simulations, we considered a fix rotation angle and same outcomes for *z*-basis measurements. Also, we did not consider extra single-qubit logic operations during the protocol, such as the *intelligent evolution* in Ref.^47^. The advantage of that work is that QZD is considered for a system of two qubits only and the protocol consists of only iterations without any final *z*-basis measurements. However in the present work and similarly in the recent work on non-local activation of bound entanglement^48^, QZD is considered only for some particles of a larger system. In these two *non-intelligent* schemes, a final *z*-basis measurement on the particles subject to QZD is required, and the protocol is successful only in some particular outcomes of the final measurement. It would be interesting as a future work to design a general framework for intelligent evolution during QZD such that once the QZD iterations are completed, the corresponding qubits can simply be discarded (without requiring final measurements), leaving the target qubits in the desired state.

## Methods

In each iteration of QZD, a set of two basic operations are performed. First, the following rotation operation is applied on each of the two qubits at the repeater station,

8 $$\begin{aligned} \displaystyle R(\theta ) = \left( \begin{array}{cc} \cos \theta &{} - \sin \theta \\ \sin \theta &{} \ \ \ \cos \theta \\ \end{array} \right) , \end{aligned}$$

which is followed by the the so-called *J*-measurements, or threshold measurements on each qubit in concern, defined by the measurement operators

9 $$\begin{aligned} J_1= |i\rangle \langle i| \otimes |j\rangle \langle j|, \ \ \ \ \ J_0 = I^{\otimes 2} -J_1 \end{aligned}$$

with $i, j \in {0, 1}$ and *I* being the two-dimensional identity operator. Along with the rotation operator, *J*-measurements play the vital role in the proposed QZD scheme. Performing the *J*-measurements, the system will be found in $|i\rangle |j\rangle$ state with a small probability $\varepsilon$, and with $1-\varepsilon$ probability it is projected to the $J_0$ subspace. Setting $i=j=1$ for example, that is $J_1= |1\rangle \langle 1| \otimes |1\rangle \langle 1|$ checking if both qubits are in $|1\rangle$ state the *J*-measurement acts like a threshold detector. Wang et al. have proposed a Josephson-junction circuit with flux qubits for implementing the J-measurement^47^ which is shown in Fig.7, and can be summarized as follows. The circuit consists of two flux qubits, $A_2$ and $B_2$ each consisting of three small junctions, and another junction *X*. The transition of *X* junction from superconducting state to normal state can be triggered with a current across it being greater than a critical value, $I_c$. Depending on the state, each flux qubit contributes a current to the circuit in either up or down direction, and with an additional bias current $I_b$, the junction *X* can switch to the normal state with a non-zero voltage *V*. In other words, turning on the bias current $I_b$ and measuring the voltage *V*, it will be determined if two qubits are in $|1\rangle |1\rangle$ state or not, i.e., leaving the qubits in the $J_0$ subspace in the latter case. Please see Ref.^47^ for further details. In summary, applying the single-qubit rotations and then performing the *J*-measurement constitute a single iteration step of the QZD.

In each iteration, the state of the system evolves in the rotate-measure procedure as $\rho \rightarrow \rho ^{r} \rightarrow \rho ^{rm}$ where

10 $$\begin{aligned} \rho ^{r} = (I \otimes R(\theta )^{\otimes 2} \otimes I ) \ \rho \ (I \otimes R(\theta )^{\otimes 2} \otimes I )^{\dag }, \end{aligned}$$

and

11 $$\begin{aligned} \rho ^{rm} = {(I \otimes J_0 \otimes I) \ \rho ^{r} \ (I \otimes J_0 \otimes I)^{\dag } \over \text {Tr}[(I \otimes J_0 \otimes I) \ \rho ^{r} \ (I \otimes J_0 \otimes I)^{\dag }]}. \end{aligned}$$

After *n* iterations, the QZD process is over and similar to the circuit model computation, two qubits at the repeater are measured in $z-$basis, and according to the results of this final measurement communicated over a classical channel, one of the Pauli operators $\{I,\sigma _x, \sigma _z\}$ is applied to the qubits of Alice and Bob, leaving them not exactly in the Bell state but in the state $\rho '$ with almost a unit fidelity to a Bell state. Here, $\{i, j \}$ of $J_1$, the rotation angle $\theta$ and the number of iterations *n* are to be determined by numerical simulations for achieving the closest $\rho '$ to a maximally entangled state. Note that in each iteration for each qubit, considering a different $\theta$ could improve the performance with the drawback expanding the search space significantly. For simplicity, we fix $\theta$ for both qubits throughout the process.

For extending the above entanglement swapping procedure to a series of repeater stations, we can assume that the entanglement swapping (ES) starts from both ends and continues towards the repeater station in the middle as in Fig. 2. Therefore, although assuming that the first ES starts with maximally entangled states, the non-maximally entangled state $\rho '$ is obtained which is to be used in the next ES, creating $\rho ''$ state with a smaller fidelity to the maximally entangled state than $\rho '$. Our numerical simulation takes into account the generated non-maximally entangled state being the output of each ES as the input to the next ES.

In order to determine how close the resulting state $\rho ^r$ is to a target state $|\Psi \rangle$, we use the fidelity defined as^1^ $F(\rho ^r) = \langle \Psi | \rho ^r |\Psi \rangle$. Depending on the task, an appropriate measure can be employed. For example, if the resulting states are to be used in a metrological task, rather than the fidelity or a monotonic entanglement measure, quantum Fisher information^57^ can be preferred. As explained in the “Discussion” section, we find the negativity as an appropriate measure for evaluating the performance of the proposed scheme. The negativity of a two-qubit state $\rho$ is found by the absolute sum of its negative eigenvalues $\mu _i$ of after partial transposition $\rho ^{\Gamma _A}$ with respect to subsystem *A* as

12 $$\begin{aligned} N(\rho ) \equiv \frac{||{\rho }^{\Gamma _A}||_{1} - 1}{2}, \end{aligned}$$

where $||A||_1$ is the trace norm of the operator *A*^58^.

## Supplementary Information

Supplementary Information.

## Data Availability

All data generated or analysed during this study are included in this published article.
