# Supplementary material for: Quantum Zeno repeaters
Source: Sci Rep. 2022 Sep 12;12:15302. doi: 10.1038/s41598-022-19170-z (PMC9468345; doi:10.1038/s41598-022-19170-z)
Supplement: Supplementary file 1 — Supplementary Information. [file 41598_2022_19170_MOESM1_ESM.pdf]

Supplementary Material for 'Quantum Zeno Repeaters'

Veysel Bayrakci[1] and Fatih Ozaydin[2,1,3]

[1] *Faculty of Engineering and Natural Sciences, Isik University, Sile, Istanbul, 34980, Turkey*
[2] *Institute for International Strategy, Tokyo International University, 1-13-1 Matoba-kita, Kawagoe, Saitama 350-1197, Japan*
[3] *CERN, 1211 Geneva 23, Switzerland*

In this Supplementary Material, we provide the density matrices of the states obtained through ES over nine repeater stations, where ρ_j denotes the state after jth ES. To reveal the turning point of negativity in Fig.6, it is straightforward to obtain the negativity of each density matrix using Eq.(12) in the Methods Section of the main text. It is important to detect the turning point of negativity to show that it does not keep decreasing over a series of repeater stations but rather starts increasing after a turning point.

$$\rho_1 = \begin{pmatrix} 0.000753558 & 0.0193976 & 0.0193976 & -0.000677401 \\ 0.0193976 & 0.499319 & 0.499319 & -0.0174372 \\ 0.0193976 & 0.499319 & 0.499319 & -0.0174372 \\ -0.000677401 & -0.0174372 & -0.0174372 & 0.000608941 \end{pmatrix}, \tag{1}$$

$$\rho_2 = \begin{pmatrix} 0.500137 & 0.00196063 & 0.00196063 & 0.499992 \\ 0.00196063 & 7.68598\text{x}10^{-6} & 7.68601\text{x}10^{-6} & 0.00196006 \\ 0.00196063 & 7.68601\text{x}10^{-6} & 7.68601\text{x}10^{-6} & 0.00196006 \\ 0.499992 & 0.00196006 & 0.00196006 & 0.499848 \end{pmatrix}, \tag{2}$$

$$\rho_3 = \begin{pmatrix} 0.000913537 & 0.0213573 & 0.0213573 & -0.000661988 \\ 0.0213573 & 0.499303 & 0.499303 & -0.0154764 \\ 0.0213573 & 0.499303 & 0.499303 & -0.0154764 \\ -0.000661988 & -0.0154764 & -0.0154764 & 0.000479705 \end{pmatrix}, \tag{3}$$

$$\rho_4 = \begin{pmatrix} 0.500258 & 0.00392158 & 0.00392158 & 0.499969 \\ 0.00392158 & 0.0000307416 & 0.0000307417 & 0.00391931 \\ 0.00392158 & 0.0000307417 & 0.0000307417 & 0.00391931 \\ 0.499969 & 0.00391931 & 0.00391931 & 0.49968 \end{pmatrix}, \tag{4}$$

$$\rho_5 = \begin{pmatrix} 0.0000692054 & 0.00588032 & 0.00588193 & -0.000159162 \\ 0.00588032 & 0.499645 & 0.499782 & -0.0135238 \\ 0.00588193 & 0.499782 & 0.499919 & -0.0135275 \\ -0.000159162 & -0.0135238 & -0.0135275 & 0.000366047 \end{pmatrix}, \tag{5}$$

$$\rho_6 = \begin{pmatrix} 0.499725 & 0.00588306 & -0.0115664 & 0.499832 \\ 0.00588306 & 0.0000692589 & -0.000136167 & 0.00588432 \\ -0.0115664 & -0.000136167 & 0.000267713 & -0.0115689 \\ 0.499832 & 0.00588432 & -0.0115689 & 0.499938 \end{pmatrix}, \tag{6}$$

$$\rho_7 = \begin{pmatrix} 0.000123053 & 0.00784181 & 0.00784289 & -0.00018147 \\ 0.00784181 & 0.499736 & 0.499805 & -0.0115645 \\ 0.00784289 & 0.499805 & 0.499873 & -0.0115661 \\ -0.00018147 & -0.0115645 & -0.0115661 & 0.000267619 \end{pmatrix}, \tag{7}$$

$$\rho_8 = \begin{pmatrix} 0.499815 & 0.00784462 & -0.0096067 & 0.499846 \\ 0.00784462 & 0.000123122 & -0.000150778 & 0.00784511 \\ -0.0096067 & -0.000150778 & 0.000184646 & -0.00960729 \\ 0.499846 & 0.00784511 & -0.00960729 & 0.499877 \end{pmatrix}, \tag{8}$$

$$\rho_9 = \begin{pmatrix} 0.00019229 & 0.0098035 & 0.0098035 & -0.000188386 \\ 0.0098035 & 0.499812 & 0.499812 & -0.00960447 \\ 0.0098035 & 0.499812 & 0.499812 & -0.00960447 \\ -0.000188386 & -0.00960447 & -0.00960447 & 0.000184561 \end{pmatrix}. \tag{9}$$
